# Development of Potent Forchlorfenuron Analogs and Their Cytotoxic Effect in Cancer Cell Lines

**DOI:** 10.1038/s41598-020-59824-4

**Published:** 2020-02-24

**Authors:** Kyu Kwang Kim, Rakesh K. Singh, Negar Khazan, Arif Kodza, Niloy A. Singh, Aaron Jones, Umayal Sivagnanalingam, Mary Towner, Hiroaki Itamochi, Rachael Turner, Richard G. Moore

**Affiliations:** 1 The Wilmot Cancer Institute at the University of Rochester Medical Center, Rochester, NY United States; 2 University of Albany, Albany, NY United States; 3 Iwate Medical University School of Medicine, Morioka Iwate, Japan

**Keywords:** Drug discovery and development, Chemotherapy

## Abstract

Forchlorfenuron (FCF) is a synthetic plant cytokinin widely used in agriculture to promote fruit size, that paradoxically inhibits proliferation, migration, and invasion in human cancer cell lines. FCF has also been shown to affect HIF-1α and HER2, which are both known to play a crucial role in cancer cell survival. In this study, we have developed potent FCF analogs through structural modification of FCF, coined UR214-1, UR214-7, and UR214-9. Compared to parental FCF, these analogs are more effective in decreasing viability and proliferation in both ovarian and endometrial cancer cell lines. These FCF analogs also suppress HER2 expression at a concentration lower than that of FCF. In addition, we found that treatment with either FCF or its analogs decreases the expression of human epididymis protein 4 (HE4), which is commonly upregulated in ovarian and endometrial cancers. Given the association between cancer behavior and HE4 production in gynecologic cancers, our findings may provide insight useful in the development of new treatment strategies for gynecologic cancers.

## Introduction

Malignancy of the ovary and uterus is relatively common and unfortunately quite lethal. The fourth most common cancer in women, uterine cancer represents approximately 60,000 new cancer diagnoses and 10,000 cancer deaths per year in the US^1^. While less common, ovarian cancer is more fatal yet, with a 5-year survival of 46.6% (www.cdc.gov/cancer/dataviz, June 2019). Current treatment of these diseases relies on a combination of surgical and medical management. While evolving, the foundation of first-line medical treatment for both is a combination of platinum-based and taxane drugs and has been so for over a decade.

Diaryl urea derivatives have been of great interest in medicinal chemistry and the small molecules containing a diaryl urea scaffold have exhibited a broad spectrum of biological activities including anti-inflammatory, antithrombotic and antimicrobial effects^2^. Additionally, diaryl urea derivatives such as sorafenib and regorafenib are used clinically for cancer treatment^3^. Forchlorfenuron (FCF; *N*-(2-Chloro-4-pyridyl)-*N*′-phenylurea) is a small synthetic urea derivative that is currently utilized in agriculture. FCF shows potent cytokinin activity and has been used worldwide as a plant fertilizer that increases fruit size. Interestingly, FCF has been shown to inhibit proliferation, anchorage independent growth, migration and invasion of cancer cell lines^4–6^. The effect of FCF has been demonstrated in various cancer types, including prostate, mesothelioma, lung, colon, breast, ovary and cervix^4^. FCF was also found to be effective in a mouse model, in which tumor growth was inhibited^4^. At a molecular level, FCF treatment causes the suppression of HIF-1α and HER2, both of which are known to be associated with more malignant cancer phenotypes^6,7^.

In this study, we have conducted a focused, structure-activity relationship study to optimize the chemical structure of FCF, with the goal of developing potent FCF analogs that could be tested for cytotoxic activity against a panel of gynecologic cancer cell lines. Ultimately, we hope such a compound could potentially be utilized as a future research tool. We also explored whether FCF or FCF analogs contribute to human epididymis protein 4 (HE4) secretion, which has been linked to the progression of ovarian cancer as well as poor patient outcomes, including advanced disease and decreased survival^8^. Finally, we delve into the role of FCF analogs on cancer cell growth factor receptor expression.

## Results

### Treatment of FCF reduced the viability of endometrial and ovarian cancer cell lines

**Figure 1:**
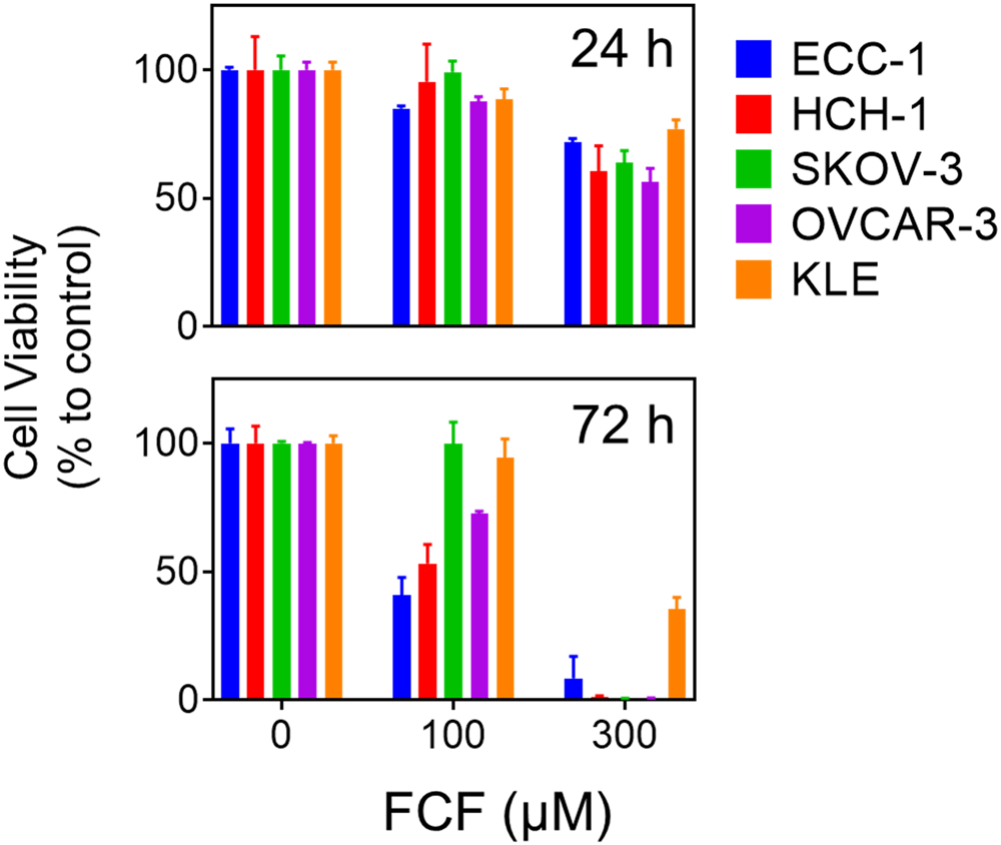
The effect of FCF on the viability of ovarian and endometrial cancer cell lines. Cancer cell lines were treated with FCF (0, 100, 300 µM) for either 24 h or 72 h, after which the cell viability was measured by MTS assay.

We first tested the effect of FCF on cellular viability in a panel of gynecologic cancer cell lines employing endometrial (ECC-1 and KLE) and ovarian (HCH-1, OVCAR-3, and SKOV-3) cancer cell lines. We observed that FCF treatment reduced the viability of these cells in a time- and dose-dependent manner (Fig. 1). Although FCF exhibited noticeable cytotoxic activity against a panel of cell lines, such an inhibition was only achieved at relatively high concentrations (at or above 100 µM).

### Structural modification of FCF

**Figure 2:**
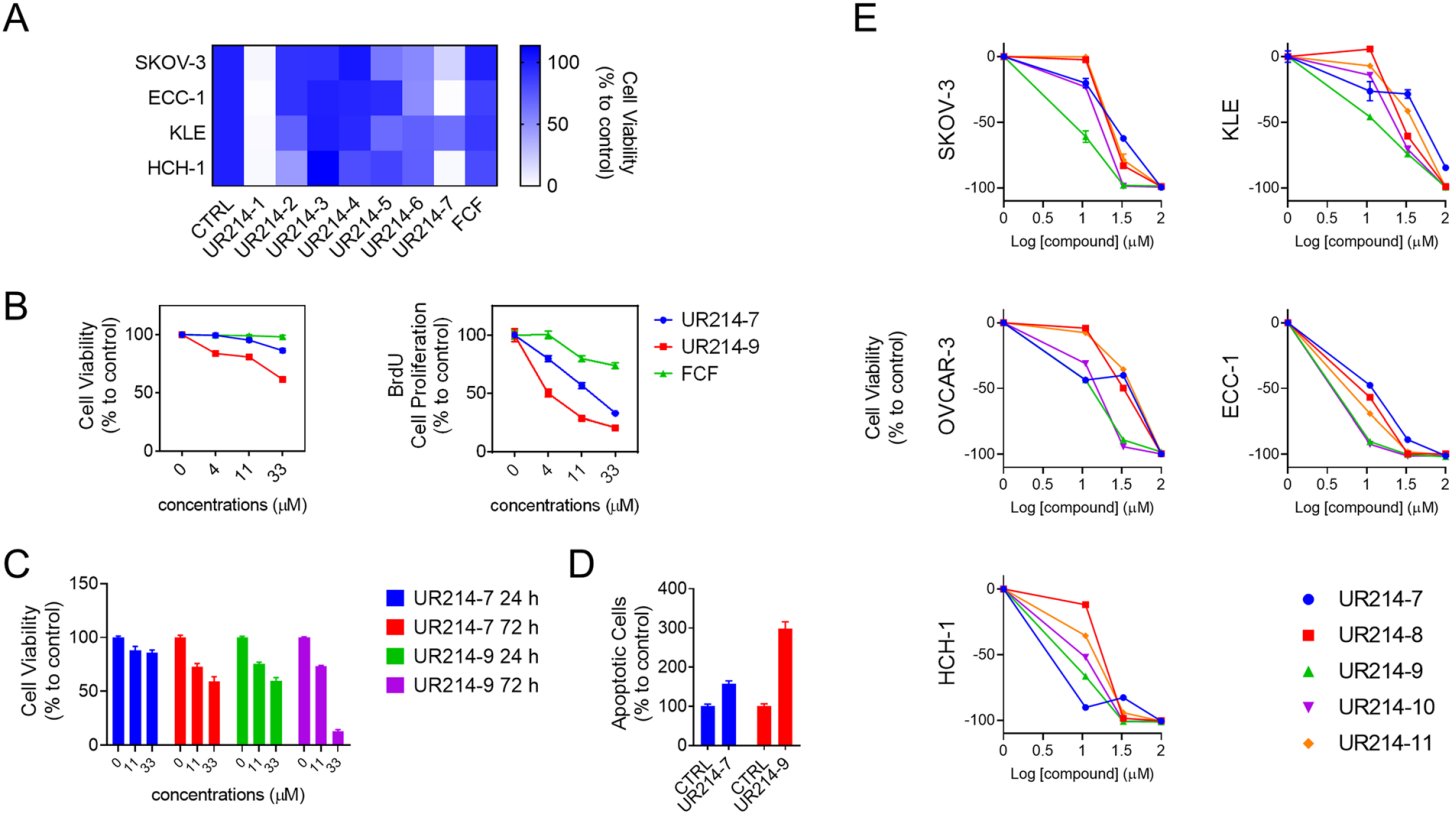
The effect of FCF analogs on the viability of ovarian and endometrial cancer cell lines. (**A**) Cell lines were treated with a fixed concentration (100 µM) of FCF or FCF analog for 24 h. After treatment, cell viability was determined by MTS assay. (*N* = 3, see Suppl. Fig. A for mean values and SEM) (**B**) ECC-1 cells were treated with UR214-7 or UR214-9 at the listed concentrations for 48 h. After treatment, cell viability was determined by MTS assay (left). Cell proliferation was measured by BrdU incorporation (right). (**C**) ECC-1 cells were treated with vehicle, UR214-7, or UR214-9 at the indicative concentrations for 24 or 72 h. Cell viability was determined by MTS assay. (**D**) ECC-1 cells were treated with UR214-7 or UR214-9 at 33 µM for 48 h. Cells undergoing apoptosis were measured by caspase-3/7 activity assay (**E**) Each indicated cells were treated with various concentrations of FCF analogs for 72 h. After treatment, cell viability was determined by MTS assay.

We conducted a systematic structural modification of the FCF scaffold with the intent of generating a highly potent FCF analog (Table 1). In this limited exercise, the substituents on the phenyl moiety were varied initially, keeping the 2-cholopyridine constant (except for UR214-5 and UR214-6). The substituents were varied to read the effect of modification on the anti-proliferative activity of the molecules. As described in Fig. 2A, substitutions such a benzyloxy (UR214-2), pyrimidinyl (UR214-4) and pyrrolyl (UR214-3) were not tolerated. However, CF_3_S substitution (UR214-1) on the phenyl ring of FCF generated very potent cytotoxic activity. The importance of 2-choloropyridine was demonstrated by replacing 2-choloropyridine with 2-chloropyrimidine while retaining the CF_3_S-Ph moiety (UR214-6); this alteration resulted in lack of activity. UR214-1 emerged as the most potent molecule against cancer cells, followed by UR214-7 (Fig. 2A). UR214-1 was not further altered, but UR214-7 was chosen for further optimization in an effort to enhance efficacy.

**Table 1:** The structure of FCF and its newly derived analogs.

Name	Structure	Name	Structure
FCF		UR214-6	
UR214-1		UR214-7	
UR214-2		UR214-8	
UR214-3		UR214-9	
UR214-4		UR214-10	
UR214-5		UR214-11

### Effects of FCF analogs on cell viability and proliferation

We elaborated UR214-7’s structure further to develop molecules UR214-8 through UR214-11. The dose-dependent effects of these FCF analogs (UR214-7 to −11) on the viability of tumor cells was tested using MTS assay (Fig. 2E). Treatment with compounds UR214-7 to −11 exerted potent anti-viability effects against ovarian (SKOV-3, OVCAR-3, and HCH-1) and endometrial (ECC-1 and KLE) cancer cells. To produce the most active compound of the series (UR214-9), we installed a chloride at the 6^th^ carbon of pyridine. UR214-9 showed potent activity across most cell lines, except for HCH-1, where UR214-7 had superior efficacy for unclear reasons (Fig. 2E). For the most part, halide substituents on the pyridine were well tolerated. Surprisingly, the replacement of 2-chloro with 2-bromo was tolerated and as such, UR214-11 did not lose activity, keeping the window open for further modifications. Next, we employed a BrdU assay to measure the anti-proliferative activity of UR214-7 and UR214-9 against ECC-1 cells at lower dose ranges. In this assay, FCF was utilized as a positive control. As shown in Fig. 2B, UR214-9 treatment for 48 hours inhibited viability and proliferation around 33 and 4 µM, respectively. UR214-7 did not alter tumor cell viability below 11 µM, but did reduce proliferation (Fig. 2B, right). In both assays, FCF lacked activity. Next, we analyzed the ability of UR214-7 and UR214-9 to induce apoptosis of ECC-1 cells. As shown in Fig. 2D, treatment with both UR214-7 and UR214-9 caused apoptosis, but the latter was much more effective. The effects of UR214-7 and UR214-9 treatment were time- and dose-dependent (Fig. 2C).

### FCF analogs selectively inhibit HER2 expression

**Figure 3:**
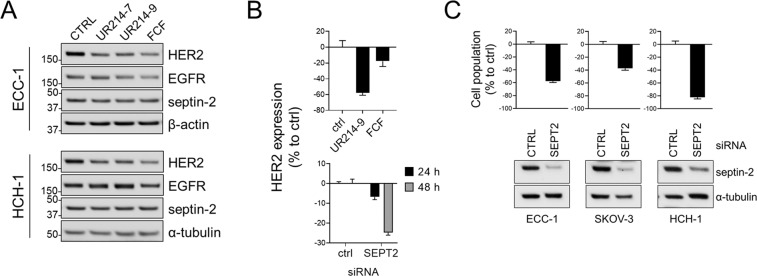
The effect of FCF analogs on HER2 expression. (**A**) ECC-1 or HCH-1 cells were incubated with UR214-7 (33 µM), UR214-9 (33 µM) or FCF (300 µM) for 24 h. The levels of each protein were determined by Western blot analysis. (**B**) HCH-1 cells were incubated with UR14-9 (10 µM) or FCF (100 µM) for 48 h (top) or transfected with septin-2 targeting siRNA or non-targeting control siRNA for 24 or 48 h (bottom). Cell lysates were collected. The cellular levels of HER2 were determined by human HER2 enzyme-linked immunosorbent assay. (**C**) The cells were transfected with septin-2 targeting siRNA or non-targeting control siRNA. At 48 h post transfection, cell population and relative expression of septin-2 were determined by sulforhodamine B assay (top) and Western blot analysis (bottom), respectively.

FCF has been shown to downregulate HER2, the cell membrane-anchored growth factor implicated in the aggressiveness of several cancer types^7^. Therefore, we sought to explore whether our newly generated FCF analogs impact HER2 expression in cancer cell lines. Treatment with UR214-7 and UR214-9 did not have an effect on EGFR expression. However, treatment with UR214-7 and UR214-9 led to the inhibition of HER2 expression in both ECC-1 and HCH-1 cells, as did FCF at a significantly higher dose (Fig. 3A). Previous study suggests that the effect of FCF on HER2 could be achieved through the disruption of septins and septin-2 knockdown decreased HER2 expression^7^. Thus, we knocked down septin-2 and determined its effect on HER2 levels. Septin-2 knockdown resulted in HER2 downregulation (Fig. 3B). Inhibition of septin-2 also decreased the viability of cancer cell lines (Fig. 3C).

### FCF analogs decrease HE4 secretion

**Figure 4:**
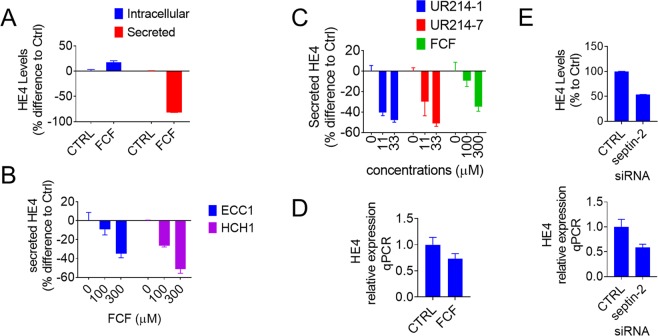
The effect of FCF analogs on the expression of HE4. (**A**) HE4 overexpression clones (OVCAR8-C5) were treated with or without FCF (300 µM) for 7 h, after which the cell lysates and matching culture media were collected and analyzed for the levels of HE4 using enzyme immunoassays. The amount of secreted HE4 was normalized to the protein concentration of respective cell lysate. (**B**) ECC-1 and HCH-1 cells were treated with the indicated concentrations of FCF for 5 h and the secreted levels of HE4 were measured. (**C**) ECC-1 cells were treated with either UR214-1, UR214-7 or FCF at the concentrations indicated for 5 h at which point the secreted levels of HE4 were determined. (**D**) ECC-1 cells were washed and incubated with FCF (300 µM) for 5 h in basal media. Relative HE4 expression was determined by real time PCR (normalized to B2M). (**E**) ECC-1 cells were transfected with septin-2 targeting siRNA or non-targeting control siRNA for 48 h (bottom) or for 72 h (top). Relative gene expression of HE4 was determined by real time PCR (normalized to TBP; bottom). The intracellular levels of HE4 were measured using enzyme immunoassays (top).

HE4 is a small secretory glycoprotein that is highly upregulated in patients with ovarian and endometrial cancers^9,10^ and is associated with malignant phenotypes of cancer^8^. Therefore, we sought to determine whether FCF or its potent analog, can impact the secretion of HE4. As shown in Fig. 4A, FCF treatment (300 µM for 7 h) resulted in a decrease in HE4 secretion by OVCAR8-C5 that stably overexpresses HE4, along with a marginal increase in intracellular levels of HE4. This effect was found to be dose-dependent in both ECC-1 and HCH-1 cells (Fig. 4B). Compared to FCF, similar inhibition of HE4 secretion was achieved by UR214-1 and UR214-7 at much lower concentrations (<10- to 27-fold; Fig. 4C). FCF treatment was found to suppress HE4 mRNA to some extent (Fig. 4D). Septin-2 disruption by small interfering RNA also resulted in reduction of HE4 expression (Fig. 4E).

### Septin overexpression is associated with increased cancer mortality

**Figure 5:**
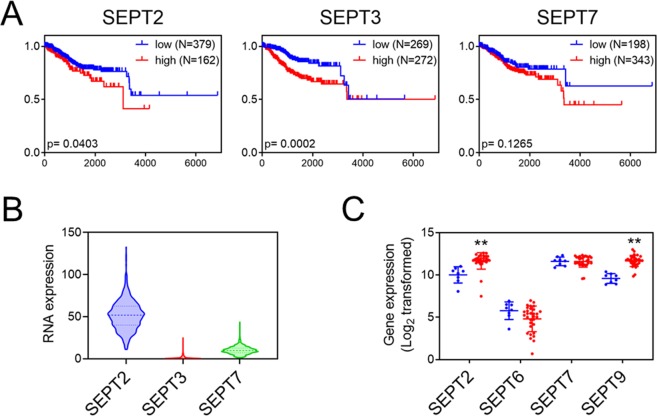
(**A**) Correlation of septins with survival rates of endometrial cancer patients was analyzed using the TCGA dataset. (*p* < 0.05); x-axis: days; y-axis: survival fractions. (**B**) Relative expression of septin-2, -3 and -7 in endometrial cancers (TCGA dataset). (**C**) Relative gene expression of various septins was analyzed from the dataset (Wong-77-MAS5.0-u133p2), which contains gene expression profiling of microdissected cancer stroma samples from high grade serous ovarian cancer patients (red) and those from normal ovarian stroma (blue). (**p < 0.01, Student’s *t*-test vs. microdissec*t*ed normal ovarian stroma).

In the present study, septin-2 knockdown caused a decrease in HER2 and HE4 expression and inhibited the viability of cancer cell lines. In addition, septins are associated with malignant cancer phenotypes^5,11–15^. Therefore, we wondered if septin expression is associated with patient outcomes in endometrial and ovarian cancers. We conducted Kaplan-Meier survival analyses for endometrial cancer stratified by expression of septin-2, -3, and -7. These septins are of particular interest because they were used for the binding study of FCF^16^. Analyses of The Cancer Genome Atlas (TCGA) (Fig. 5A) show that both septin-2 and septin-3 overexpression correlate with increased mortality in endometrial cancer. In endometrial cancer, relative expression of septin-2 was highest, followed by septin-7. Septin-3 expression was found to be very low (Fig. 5B). The correlation between septin-2 expression and increased mortality was also found in kidney, lung, liver, and pancreatic cancers, but not in ovarian cancer (data not shown). Cancer-associated fibroblasts (CAFs) are found in the stroma that surrounds and supports cancer cells. The septin network is known to support CAFs in creating a pro-tumorigenic microenvironment^17^. Therefore, data that profiled septin expression in ovarian cancer-associated vs. normal ovarian stroma were also analyzed (Wong-77-MAS5.0-u133p2). In cancer-associated stroma, the expression of septin-2 and septin-9 was significantly upregulated, while septin-6 and septin-7 were unchanged (Fig. 5C).

## Methods

### Cell lines, cell culture and reagents

HCH-1 cell line was kindly provided by Dr. Hiroaki Itamochi (Iwate Medical University, Japan). All other cell lines were purchased from the American Type Culture Collection. OVCAR8-C5 was previously developed^18^. The cells were maintained in either DMEM (SKOV-3, KLE, OVCAR-3 OVCAR8-C5 and IGROV-1) or RPMI-1640 (ECC-1 and HCH-1), supplemented with 10% fetal calf serum (or 20% for OVCAR-3), penicillin (100 units/mL), and streptomycin (100 µg/mL) at 37 °C with 5% CO_2_ in a humidified incubator. Antibodies were purchased from Abcam (septin-2; ab179436) and Cell Signaling Technology; ERK (9102), p-ERK (4370), β-Actin (3700), α-Tubulin (2144), EGFR (4267) and HER2 (4290). FCF was purchased from Abcam. All other chemicals were from Sigma Aldrich.

### Synthesis of FCF analogs

FCF analogs were synthesized by coupling variously substituted aryl isocyanates with differentially substituted 4-aminopyridines in (0.1:0.1) molar ratio in dry DMF at 65 °C overnight. The reaction was monitored using thin-layer chromatography plates with DCM-MeOH or pure ethyl acetate as eluent. Spots were monitored in a UV chamber. The reaction mixture, upon completion of the reaction, was poured into wet ice mixture and triturated, then the separated solid was filtered by vacuum. The product was washed with hexane, followed by diethyl ether, and was dried under vacuum. The compounds were characterized by spectrometric techniques.

### Quantitative real-time PCR

The indicated cell lines were transfected with siRNA targeting septin-2 or non-targeting control siRNA (Santa Cruz Biotechnology: sc-37007 or sc-40936) using Lipofectamine 3000 (Invitrogen). Tri reagent and Direct-zol kit (Zymo Research) were used to lyse and isolate total RNA. Reverse transcription was performed using iScript cDNA synthesis kit (BioRad), both following the manufacturer’s recommendations. Quantitative real-time PCR was conducted using QuantStudio 12 K Flex Real-Time PCR System (ABI) and the Taqman Gene Expression Assay (ABI) consisting of the FAM-labelled probes as follows: SEPT2 (Hs01565417_m1); WFDC2 (Hs00899484_m1); TBP (Hs00427620_m1) or B2M (Hs00187842_m1), which was used as reference for normalization.

### Cell viability, proliferation and apoptosis assay

Cell viability, proliferation, and cellular apoptosis were measured using the MTS (Promega), BrdU cell proliferation (Cell Signaling Technology), and Caspase-Glo 3/7 apoptosis detection (Promega) assays, following the respective manufacturer’s recommendations with suitable modification.

### Enzyme-linked immunosorbent assay (ELISA) and immunoblotting

Cells were maintained overnight in the culture media described above. The medium was removed and replaced with fresh complete medium containing either vehicle (DMSO) or FCF/FCF analogs in the indicated conditions. Following the treatment, cell lysates and supernatants were collected and subjected to HE4 assay (Human HE4 Quantikine ELISA Kit, R&D systems). The amount of HE4 in supernatant was normalized to the protein concentration of matching cell lysate. The levels of HER2 were determined by Human Total ErbB2/Her2 DuoSet IC ELISA (R&D systems). Western blot analysis was conducted using the protocol previously published^19^.

### Data acquisition and statistical analysis

Gene expression profiling of septins, comparing microdissected normal ovarian stroma (*N* = 8) against that of tumor stroma samples (*N* = 31) from high grade serous ovarian cancer patients, was obtained from Mixed Ovarian Cancer (CAFs)-Wong-77-MAS5.0-u133p2 dataset through ‘R2: Genomics Analysis and Visualization Platform (http://r2.amc.nl)’. Mean values with SD were plotted and compared using Student *t*-test (two tail, unpaired heteroscedastic *t*-test, significant difference when *p* < 0.01). The prognostic assessment of septin-2 amongst different cancers or septins in endometrial cancer was conducted using TCGA data acquired through Human Protein Atlas (http://www.proteinatlas.org)^20^. Survival curves between septin high and low population and their statistical significance were analyzed using GraphPad Prism software (Mantel-Cox test).

## Discussion

In the present study, we found that treatment with FCF reduced cell viability in ovarian and endometrial cancer cell lines in the range 100–300 µM, which is pharmacologically undesirable. Through efforts to optimize FCF’s structure, we have generated highly potent analogs, UR214-1, UR214-7 and UR214-9. Treatment with these analogs blocked proliferation in multiple endometrial and ovarian cancer cells at considerably lower doses than FCF. We found that UR214-9 can inhibit cancer cell proliferation in the range 4–5 µM. It is possible that further structural optimization could lead to a nanomolar disruptor that would then be appropriate for further animal and potentially human trials.

Septins are a class of cytoskeletal proteins that are associated with malignant phenotypes of cancer^5,11–15^. Additionally, septins have been increasingly linked to cancer oncogene expression, including EGFR, HER2 and the HIF-1α/angiogenesis axis^7,21–23^. It is therefore not surprising that we have linked high septin-2 levels to worse patient survival in several cancer types, including endometrial cancer. Additionally, ovarian tumor stroma has been found to be highly enriched for septin-2 and -9, prompting us to further investigate the role of these two structural proteins in ovarian cancer.

HE4 is a secretory glycoprotein encoded by *WFDC2*. It is found to be upregulated in ovarian and endometrial cancers^9,24^ and has been shown to increase cancer cell proliferation, migration, invasion, metastasis and chemoresistance^8^. Our findings show that treatment with UR214-1 and UR214-7 results in reduced HE4 secretion. To our knowledge, this is the first work to identify a putative HE4 inhibitor. Therefore, these FCF analogs may have particular therapeutic benefit in gynecologic cancers.

To date, FCF is the only known small molecule septin inhibitor. Septins are not found in land plants^25^ and so it would seem logical that FCF, which has profound effects on plant growth^26^, participates in cellular processes not involving septins. Indeed, non-septin effects of FCF have been described^27^. Nevertheless, FCF has also been shown to inhibit septin dynamics and disrupt the assembly of septin-based structures^28^. *In silico* studies suggest that FCF could interact with a nucleotide-binding pocket of septins^16^. In the present study, treatment with FCF analogs caused downregulation of both HER2 and HE4, which appeared to mimic the effect of septin-2 knockdown. It would be interesting to know if FCF analogs exert their activity through septin interference. Future study will be needed to investigate this.

This study was designed to develop potent FCF analogs for the purpose of testing their biological effects, with the goal of conducting an *in vivo* study in the future. The lead molecules identified have been coined UR214-1, UR214-7 and UR214-9. We expect these putative septin inhibitors hold promise in the pharmacologic treatment of cancer, given the similarities between septins and other structural proteins such as tubulin, which is the target of numerous FDA-approved cancer therapies. Animal studies evaluating the therapeutic potential of these novel agents are warranted to determine their antitumor effects and *in vivo* safety.

## Supplementary information

Supplementary information.
