# Supplementary material for: Development of Potent Forchlorfenuron Analogs and Their Cytotoxic Effect in Cancer Cell Lines
Source: Sci Rep. 2020 Feb 24;10:3241. doi: 10.1038/s41598-020-59824-4 (PMC7039965; doi:10.1038/s41598-020-59824-4)
Supplement: Supplementary file 1 — Supplementary information. [file 41598_2020_59824_MOESM1_ESM.docx]

Development of Potent Forchlorfenuron Analogs and Their Cytotoxic Effect in Cancer Cell Lines

Kyu Kwang Kim, PhD^1,*^, Rakesh K. Singh, PhD^1^, Negar Khazan, PhD^1^, Arif Kodza^1^, Niloy A. Singh^2^, Aaron Jones^1^, Umayal Sivagnanalingam^1^, Mary Towner^1^, MD, Hiroaki Itamochi, MD, PhD^3^, Rachael Turner, MD, PhD^1^, Richard G. Moore, MD^1^

1: The Wilmot Cancer Institute at the University of Rochester Medical Center, Rochester, NY, United States.

2: University of Albany, Albany, NY, United States.

3: Iwate Medical University School of Medicine, Morioka, Iwate, Japan.

*: Corresponding author


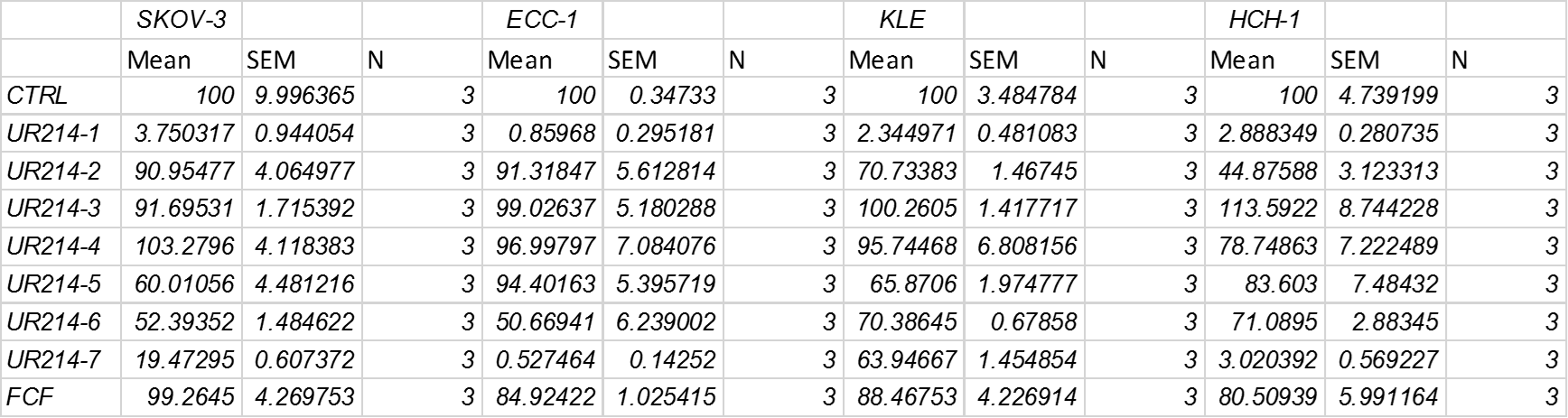


Suppl. Figure A: SKOV-3, ECC-1, KLE or HCH-1 cells were incubated in triplicate with the fixed concentration (100 µM) of indicative FCF analogs for 24 h. The cell viability was determined by the MTS assay as described in “Methods”.


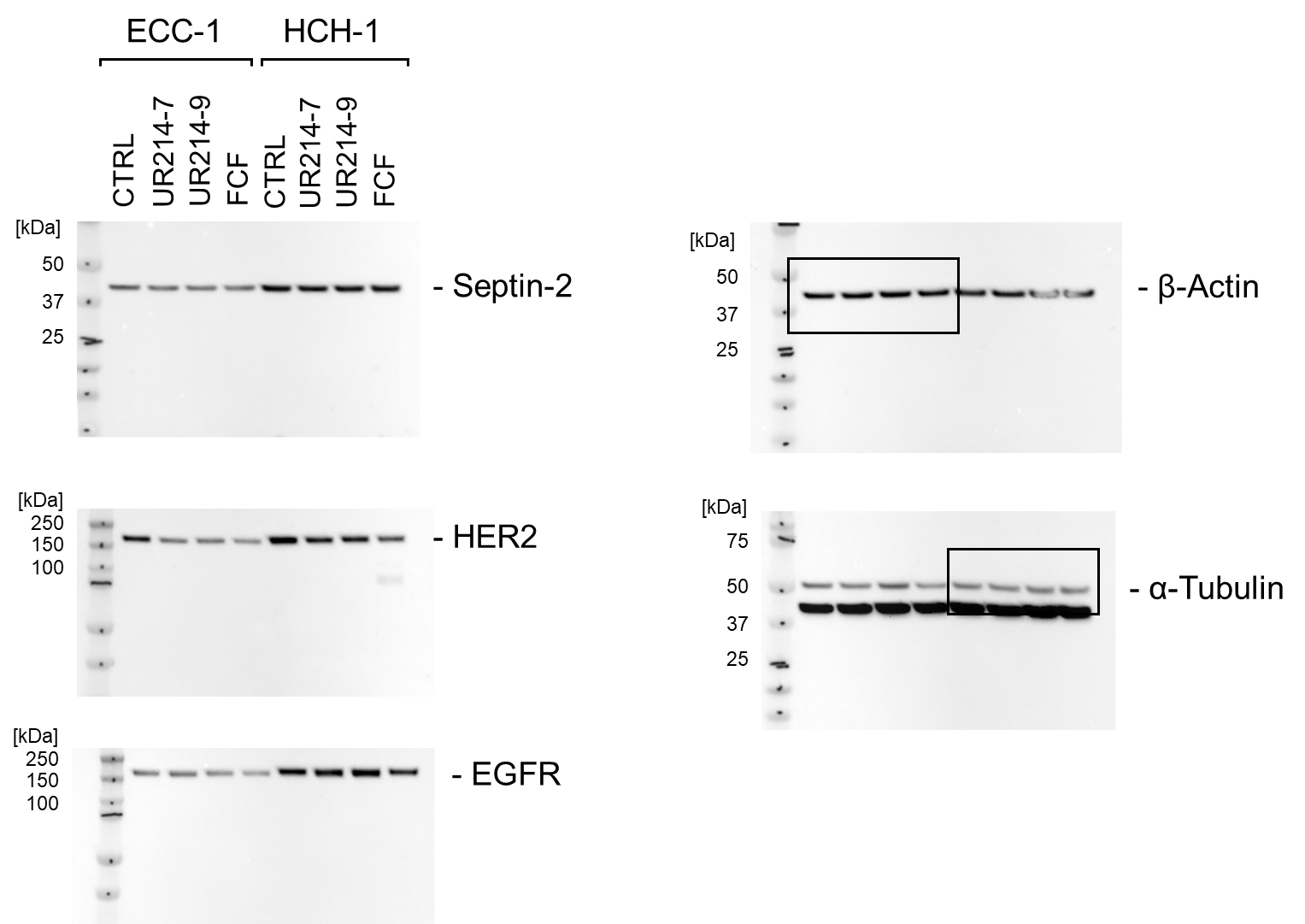


Suppl. Figure B: Images of full length gels used in Figure 3A.


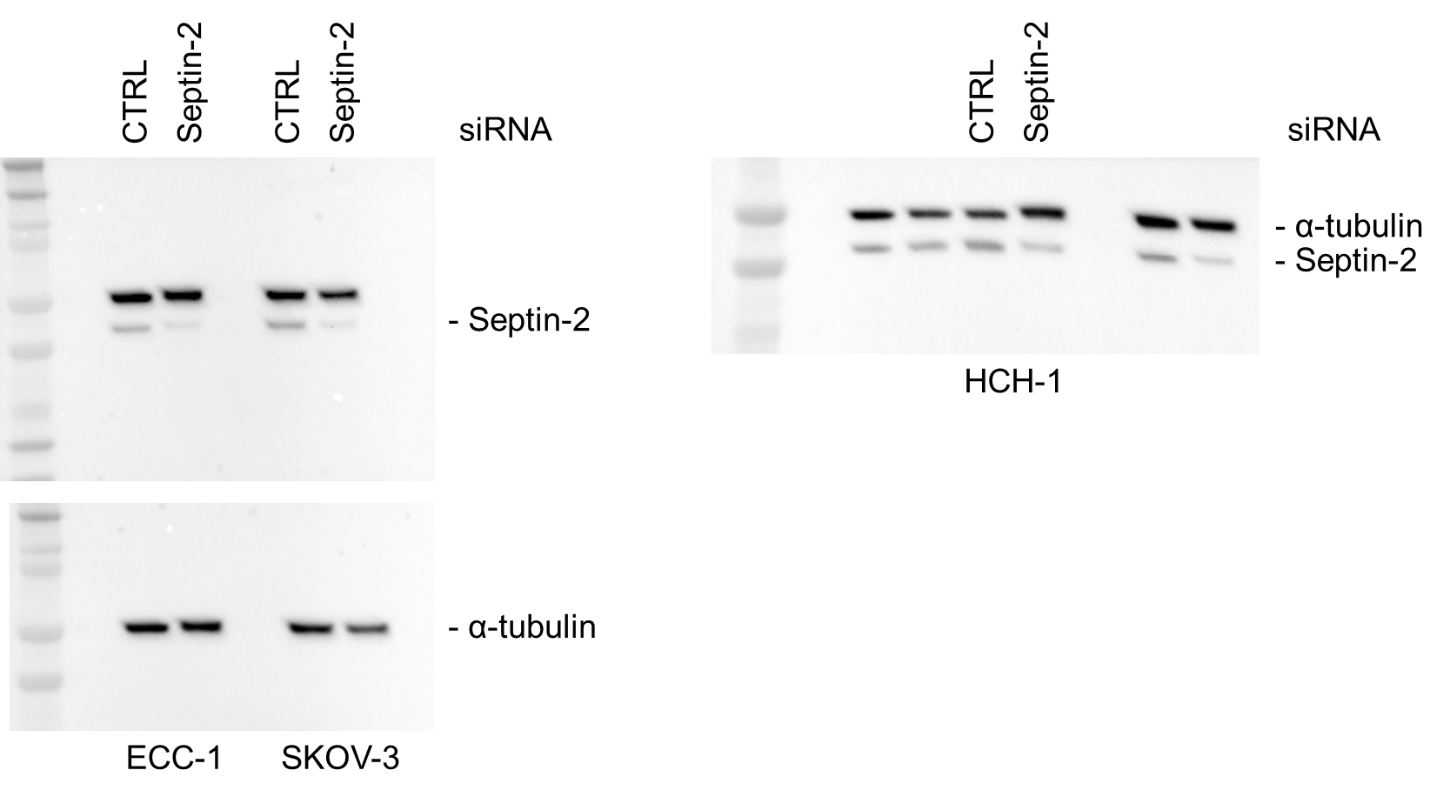


Suppl. Figure C: Images of full length gels used in Figure 3C.
